# Supplementary material for: Efficacy and safety of using auditory-motor entrainment to improve walking after stroke: a multi-site randomized controlled trial of InTandemTM
Source: Nat Commun. 2024 Feb 8;15:1081. doi: 10.1038/s41467-024-44791-5 (PMC10853163; doi:10.1038/s41467-024-44791-5)
Supplement: Supplementary file 1 — Supplementary Information [file 41467_2024_44791_MOESM1_ESM.pdf]

Supplementary Information

All study participants were provided general instructions at the start of each intervention session. The language recommended in the clinical trial protocol is as follows:

- “Please be aware that your training session will consist of 30 minutes of walking. This does include any breaks you take. However, time will not stop while you are resting.”
- “You may take standing or seated rest breaks as needed, but please know the intent of the study is for you to walk as much and as safely as possible.”
- (If applicable) "We will be monitoring your vitals while you walk, and if we feel it is necessary to have you rest, we will ask you to do so. Once it is safe for you to continue, we will tell you."
- "We will avoid conversation during the rest breaks or training, but if you need to tell us something important, please do so. For example, if you experience chest pain, dizziness, or any other need to stop, let us know immediately.”
- For InTandem subjects ONLY: "To start, you will walk at a comfortable pace. When the music begins, try your best to walk to the beat of the music."
- For Active Control subjects ONLY: “To start, you will walk at a comfortable pace."
- "Do you have any questions or concerns before we start the training?"
