# Supplementary material for: Efficacy and safety of using auditory-motor entrainment to improve walking after stroke: a multi-site randomized controlled trial of InTandemTM
Source: Nat Commun. 2024 Feb 8;15:1081. doi: 10.1038/s41467-024-44791-5 (PMC10853163; doi:10.1038/s41467-024-44791-5)
Supplement: Supplementary file 3 — Reporting Summary [file 41467_2024_44791_MOESM3_ESM.pdf]

nature portfolio

Corresponding author(s): Louis Awad

Last updated by author(s): 12/08/2023

Reporting Summary

Nature Portfolio wishes to improve the reproducibility of the work that we publish. This form provides structure for consistency and transparency in reporting. For further information on Nature Portfolio policies, see our Editorial Policies and the Editorial Policy Checklist.

Statistics

For all statistical analyses, confirm that the following items are present in the figure legend, table legend, main text, or Methods section.

n/a	Confirmed	
☐	☒	The exact sample size (*n*) for each experimental group/condition, given as a discrete number and unit of measurement
☐	☒	A statement on whether measurements were taken from distinct samples or whether the same sample was measured repeatedly
☐	☒	The statistical test(s) used AND whether they are one- or two-sided<br>*Only common tests should be described solely by name; describe more complex techniques in the Methods section.*
☐	☒	A description of all covariates tested
☐	☒	A description of any assumptions or corrections, such as tests of normality and adjustment for multiple comparisons
☐	☒	A full description of the statistical parameters including central tendency (e.g. means) or other basic estimates (e.g. regression coefficient) AND variation (e.g. standard deviation) or associated estimates of uncertainty (e.g. confidence intervals)
☐	☒	For null hypothesis testing, the test statistic (e.g. F, t, r) with confidence intervals, effect sizes, degrees of freedom and P value noted<br>*Give P values as exact values whenever suitable.*
☒	☐	For Bayesian analysis, information on the choice of priors and Markov chain Monte Carlo settings
☒	☐	For hierarchical and complex designs, identification of the appropriate level for tests and full reporting of outcomes
☒	☐	Estimates of effect sizes (e.g. Cohen's *d*, Pearson's *r*), indicating how they were calculated

Our web collection on statistics for biologists contains articles on many of the points above.

Software and code

Policy information about availability of computer code

Data collection	Data were captured using the Electronic Data Capture (EDC) system (Castor, New York, New York).
Data analysis	Data analysis was conducted using the IBM SPSS Statistics (Version 27) software.

For manuscripts utilizing custom algorithms or software that are central to the research but not yet described in published literature, software must be made available to editors and reviewers. We strongly encourage code deposition in a community repository (e.g. GitHub). See the Nature Portfolio guidelines for submitting code & software for further information.

Data

Policy information about availability of data

All manuscripts must include a data availability statement. This statement should provide the following information, where applicable:

- Accession codes, unique identifiers, or web links for publicly available datasets
- A description of any restrictions on data availability
- For clinical datasets or third party data, please ensure that the statement adheres to our policy

The processed data generated in this study are provided in the Supplementary Information/ Source Data file.

Research involving human participants, their data, or biological material

Policy information about studies with human participants or human data. See also policy information about sex, gender (identity/presentation), and sexual orientation and race, ethnicity and racism.

Reporting on sex and gender	Sex was not considered in the study design, but both sexes were recruited at equal rates. Final enrollment counts included 42 (58.3%) males and 30 (41.7%) females as reported in Table 1.
Reporting on race, ethnicity, or other socially relevant groupings	Ethnicity, race, and education level were collected for all subjects via self-report and reported in Table 1.
Population characteristics	Subjects were an average 62.3 years old and an average 8.1 years after diagnosis of stroke. 58.3% of subjects were male; 41.7% were female. Approximately 81% identified as "not hispanic or latino", whereas 10% identified as "hispanic or latino". 43% identified as Black or African American, 43% identified as White, 1.4% identified as Asian, 2.8% identified as more than 1 race, and 2.8% identified as Unknown, and 6.9% were not reported. The majority of subjects had some college education (34.7%), 29.2% were college graduates, 18.1% were high school graduates, 12.5% had graduate degrees, and 5.6% had some high school education.
Recruitment	The multi-site, prospective, interventional, 2-arm randomized controlled trial (NCT04121754) of the safety and efficacy of the InTandem neurorehabilitation system enrolled 87 participants between September 2019 and January 2022. Recruitment occurred across 8 US rehabilitation centers from existing patient research registries, word of mouth, and recruitment efforts (including flyers and support groups). Participants were compensated for their time.
Ethics oversight	The trial was approved by the Institutional Review Boards for each participating center as follows: Advarra for Carolinas Medical Center, Boston University Charles River Campus IRB (which oversaw activities at both Boston University and Spaulding Rehabilitation Hospital), IRB of the Icahn School of Medicine at Mount Sinai, Johns Hopkins Medicine IRB, Kessler Foundation IRB, Northwestern University IRB, and UNC at Chapel Hill Non-Biomedical IRB. Written informed consent was secured for all participants. All procedures were in accordance with institutional guidelines.

Note that full information on the approval of the study protocol must also be provided in the manuscript.

Field-specific reporting

Please select the one below that is the best fit for your research. If you are not sure, read the appropriate sections before making your selection.

☒ Life sciences ☐ Behavioural & social sciences ☐ Ecological, evolutionary & environmental sciences

For a reference copy of the document with all sections, see nature.com/documents/nr-reporting-summary-flat.pdf

Life sciences study design

All studies must disclose on these points even when the disclosure is negative.

Sample size	For the study primary endpoint of a change in gait speed overtime between groups, this study was originally powered (power = .80) to detect a moderately large to large effect (d =.64 to .82) for a plausible range of Intraclass correlation coefficients (ICCs), (r = .05 to r = .20), and conservative estimates of the correlation among repeated measures (r =.4 to r = .6) for n=66 and alpha= 0.05.
Data exclusions	The trial was disrupted by the COVID-19 PHE. According to guidance from FDA, the impact of COVID-19 on the trial data was examined by assessing the comparability of participants enrolled before versus during the COVID-19 PHE. The investigation revealed an effect of COVID-19 that was reasonably explained by a non-random change in trial management at one center, and ultimately required administrative removal of 8 individuals to resolve the observed effect of COVID-19. More specifically, 8 individuals (4 from the experimental group and 4 from the control group) were found to have been recruited into the trial during the COVID-19 PHE shortly after first completing another walking intervention trial. The washout period imposed between the two competing trials was discovered to be inconsistent with the precedent set in the InTandemTM trial protocol for a washout period. This deviated recruitment strategy during the COVID-19 PHE was considered an unanticipated event that affected only these eight participants; their administrative removal resulted in COVID-19 no longer affecting the trial data.
Replication	Replication was not carried out for this RCT. However, this RCT does replicate previous findings from feasibility studies (see Introduction)
Randomization	Study participants were randomized using the EDC.
Blinding	Blinding is not possible for this walking rehabilitation clinical trial given the nature of the intervention (i.e., participants walk with music)

Reporting for specific materials, systems and methods

We require information from authors about some types of materials, experimental systems and methods used in many studies. Here, indicate whether each material, system or method listed is relevant to your study. If you are not sure if a list item applies to your research, read the appropriate section before selecting a response.

Materials & experimental systems

n/a	Involved in the study
☒	☐ Antibodies
☒	☐ Eukaryotic cell lines
☒	☐ Palaeontology and archaeology
☒	☐ Animals and other organisms
☐	☒ Clinical data
☒	☐ Dual use research of concern
☒	☐ Plants

Methods

n/a	Involved in the study
☒	☐ ChIP-seq
☒	☐ Flow cytometry
☒	☐ MRI-based neuroimaging

Clinical data

Policy information about clinical studies

All manuscripts should comply with the ICMJE guidelines for publication of clinical research and a completed CONSORT checklist must be included with all submissions.

Clinical trial registration: NCT04121754

Study protocol: The study protocol is added as an appendix in supplementary material with the manuscript

Data collection: The multi-site, prospective, interventional, 2-arm randomized controlled trial (NCT04121754) of the safety and efficacy of the InTandem neurorehabilitation system enrolled 87 participants between September 2019 and January 2022. Eight US Rehabilitation Centers participated in this study (see Manuscript). All intervention sessions occurred on an overground track or hallway of at least 100 feet in length. In addition, the space used for intervention sessions could not have any discernible background music or audible distractions that could compete with the auditory intervention. Though no cueing or walking instructions were provided to study participants during training, all study participants were provided general instructions at the start of each intervention session (see Appendix A for the language recommended in the clinical trial protocol).

Outcomes: The 10-meter walk test (10mWT; comfortable speed) was used to measure the trial's primary endpoint and was conducted at the baseline screening, the trial closing visit, and to start and end each intervention session. AEs were collected at each trial visit, or volunteered by the participant between trial visits, and captured on a standard form. The standard AE form collected a description of the event, the onset and resolution dates (or if the event was ongoing), the severity, management/treatment, outcome, and determination of the relationship to the intervention.
